# Barriers to using personal protective equipment by healthcare staff during the COVID-19 outbreak in China

**DOI:** 10.1097/MD.0000000000023310

**Published:** 2020-11-25

**Authors:** Jing Fan, Ying Jiang, Kaihui Hu, Xiao Chen, Qian Xu, Yujiao Qi, Hubin Yin, Xin Gou, Simin Liang

**Affiliations:** a Department of Urology; b Department of Gynecology, the First Affiliated Hospital of Chongqing Medical University, Chongqing, China.

**Keywords:** COVID-19, health care personnel, personal protective equipment

## Abstract

The spread of coronavirus disease 2019 (COVID-19) around the world has put a heavy burden on human society and is also a great challenge facing medical staff. This study aimed to assess the difficulties faced by health care personnel (HCP) in using personal protective equipment (PPE) in clinical practice during the COVID-19 outbreak in Wuhan, China. One hundred twenty medical staff from the First Affiliated Hospital of Chongqing Medical University presented to the Wuhan First Hospital to provide medical assistance, from whom 20 HCP volunteered to participate in a focus group discussion attended by infection control nurse leaders. Participants’ responses and discussions were recorded, and the content was analyzed for themes. Observed difficulties included inappropriate PPE sizes, the design of the PPE and its complexity of use, doubts related to the quality and effectiveness of PPE, potential risks during doffing, space layout between clean and contaminated area, and poor comfort with PPE use. Other factors, such as the support environment, management, processes, preparedness, HCP, and equipment can also have a positive or negative impact on the use of PPE. Future efforts to optimize PPE use should focus on strengthening training for HCP using real items for increasing compliance with standardized protocols, improving PPE design, and performing further research on the risks, benefits, and best practices of PPE use.

## 1 Introduction

A novel coronavirus, first identified in Wuhan, Hubei province, China at the end of 2019, caused an outbreak of acute infectious pneumonia in China and many other countries, such as United States, United Kingdom, France, Italy, Korea, Japan, Thailand, and Singapore. Recently, the disease was formally named coronavirus disease 2019 (COVID-19) by the World Health Organization and the virus was named Severe Acute Respiratory Syndrome (SARS)-CoV-2 by the International Committee on Taxonomy of Viruses. The number of cases of COVID-19 has surpassed the number of cases of SARS that occurred in 2003.^[1,2]^

The Chinese government and health care personnel (HCP) have taken rapid and efficient measures to control the spread and worsening of the epidemic, and the prevention and control of COVID-19 has been critical. Personal protective equipment (PPE) is the most powerful protection provided by the Chinese government or donated by the public to protect HCP from contact with infectious agents. PPE includes gowns, surgical caps, masks, respirators, coveralls, gloves, goggles, and shoe covers. Unfortunately, risk of infection remains for HCP equipped with protective clothing if the PPE apparel is improperly used.^[3]^ Our study aimed to assess the difficulties in PPE use found in the actual work of HCP in Wuhan First Hospital during the COVID-19 outbreak.

## 2 Methods

A focus group discussion was performed on February 25, 2020 with infection control nurse leaders from the First Affiliated Hospital of Chongqing Medical University, and this study was approved by the Ethics Committee of the First Affiliated Hospital of Chongqing Medical University and is based on the ethical principles of medical research involving human objects in the Helsinki Declaration.

To better fight the epidemic, 120 HCP from the First Affiliated Hospital of Chongqing Medical University volunteered to go to the Wuhan First Hospital to provide medical assistance. Purposive sampling was used to recruit HCP who met these criteria: had over 2 weeks of work experience in managing COVID-19 patients, voluntarily agreed to participate in the study. Of the 43 HCP who met these criteria, over half refused participation due to lack of interest (n = 8) or busy work (n = 15). Therefore, 20 HCP were included finally in the focus group discussion. All participants signed an informed consent form before the study.

Information was collected and integrated in semistructured interview conducted in the meeting room of the hospital. Participants were asked to describe their experiences, concerns, or difficulties in using PPE. Their comments and discussions were recorded in detail, and the content was summarized into 6 themes according to the mentioned frequency. We also produced a mind map to summarize the potential difficulties affecting PPE use.^[4]^ All interviews were tape recorded with the participants’ permission. Each discussion lasted 60 to 90 minutes.

## 3 Results

### 3.1 Participants’ characteristics

Thirteen of the participants were women, with a mean age of 28.7 years old (range, 24–49), and 7 were man, with a mean age of 32.3 years old (range, 27–52). Ten of the participants had a bachelor's degree, 6 had a master's degree, and 4 had a doctor's degree. The participants had worked as HCP on average for 8.5 years (range, 2–29). Eight of them had work experiences in surgical wards, and 12 of them were working in medical wards.

### 3.2 Difficulties in PPE use

Difficulties mentioned by the participants were classified into 6 themes: inappropriate PPE size, design of PPE and complexity of use, doubts related to the quality and effectiveness of PPE, potential risks during doffing, space layout between clean and contaminated area, as well as poor comfort of PPE use. In addition, other barriers such as inadequate supervision, lack of practical training, customization of PPE programs, and PPE variations were also worthy of attention.

#### 3.2.1 Theme 1: Inappropriate PPE size

Due to the limited sizes available, 1-piece coverall sizing did not accommodate different body proportions well. For instance, petite females wearing baggy coveralls felt that the PPE blunted their work. Excess material increased contamination risk resulting from dragging it across surfaces, the presence of excess folds and an increased tripping hazard that made it more difficult to function. More importantly, improper sizing of PPE could hinder nursing procedures and even puncture procedures and increase the risk of occupational exposure. For instance, 1 nurse suffered a needle stick wound while collecting venous blood from a confirmed COVID-19 patient due to oversized gloves. Likewise, gaps often occurred in the chin area of small-jawed female HCP wearing N95 respirators.

#### 3.2.2 Theme 2: Design of PPE and complexity of use

PPE design does not enable easy distinction between the contaminated and clean sides of the items. It is difficult for HCP to distinguish between safety and danger, increasing their risk for contaminating clean areas of PPE while they were taking off contaminated gloves. In addition, some areas were not well covered by PPE, for instance, gowns were often too large and left parts of the neck exposed, even when sized appropriately. Participants’ backs occasionally became exposed during work. Extended cuff gloves often did not stay secured, leaving the skin around the wrist area exposed.

The core components of currently recommended PPE against COVID-19 require covering all parts of the body using head/neck covers and boots or closed shoes. As the number of PPE items increased, the doffing process involving multiple steps generally rendered the use of PPE more tedious and difficult. For example, the outer gloves were not the first doffing item when the outer shoe cover ropes were untied first. Sometimes the overlapping parts of different PPE items were inadequate, such as the gaps between an N95 respirator and safety goggles, and using separate items for the head covering and face shield may expose the forehead.

#### 3.2.3 Theme 3: Doubts related to the quality and effectiveness of PPE

The use of PPE in high-risk environments is different, in part due to insufficient evidence on the effectiveness of PPE. The impermeability and tightness of the covering, including connections between various parts of PPE, is a common concern. Some HCP described that they found it difficult to assess the impermeability ratings and described the body covering as a similar impervious material but without a logo or any hints. Three participants (15%) also wanted to obtain valid evidence of the duration of effectiveness of the PPE and the optimal frequency of replacement.

#### 3.2.4 Theme 4: Potential risks during doffing

When unzipping, the front of the coverall often curled inward, making contact with the healthcare workers’ scrubs and neck. Unzipping the coverall's zipper caused gloves to get stuck or tear. Similarly, when unsealing the flap that covers the zipper of a coverall, the gloves stick to the adhesive and cause the possibility of tearing. Taking off the coverall requires a certain amount of force, which creates a risk of splash pollution.

It is particularly difficult to take off the second layer of gloves as they tended to stick to the first layer. HCP found it hard to distinguish which layer of gloves they were currently doffing as the gloves were usually of the same color. Due to the movement of the wrist during nursing processes, the gloves were detached from PPE, especially when wearing gloves of improper size. Outer gloves can be adjusted, while inner gloves are not easy to adjust. After removing the outer gloves, the wrist skin was occasionally exposed, increasing the risk of infection.

All shoe covers were difficult to remove, especially above the heels, and nonintegral shoe covers (i.e., shoe covers worn separately from gowns/coveralls) were particularly difficult. The shoes worn by the participant under the shoe cover also affected doffing. In some cases, slippers were partly removed when doffing shoe covers, while larger running shoes fit better.

Another error in removing PPE included improper mask removal. Sometimes, taking off the respirator after doffing a body covering may expose a clean inner layer to the outermost layer, the face shield. Furthermore, donning/doffing often requires assistance, increasing the risk of cross-contamination for HCP. We summarized several common problems related to doffing PPE (Table 1).

**Table 1:** Doffing experiences reported by HCP.

Problems	Number (%) (n = 20)
Experience any problem in doffing?		
Yes	7 (35%)
No	13 (65%)
Which PPE item was most bothersome to remove?	
Coveralls	9 (45%)
Gowns	7 (35%)
Shoe covers	6 (30%)
N95 respirators	5 (25%)
Gloves	3 (15%)
Goggles	1 (5%)

#### 3.2.5 Theme 5: Space layout between clean and contaminated area

Participants also discussed how the space layout influenced their ability to follow PPE protocols. It seems that the areas considered clean and contaminated varied across different HCP. In fact, the rules pointed that red zone was within the wards and outside corridors were considered clean, but some individuals did not follow the rules strictly. In some departments, lockers known as “Only for HCP” were located just outside the patient's room due to limited space in the ward. Although this area required HCP to remove all PPE before entering, some continued to wear their equipment outside the room.

Placing a single piece coverall into a disposal bin was a challenge because of the large volume of material. Coveralls often fell to the ground, potentially polluting the environment and HCP in the room. Disposal bins were quickly filled, resulting in contaminated items overflowing, sometimes into “clean” areas.

#### 3.2.6 Theme 6: Poor comfort with PPE use

HCP mentioned overheating in various forms of PPE, usually after wearing coveralls for a short period. They described heat as a major factor affecting comfort during PPE use. Working in impermeable coveralls was reportedly difficult, as workers wearing PPE sweated profusely, even with minimal exertion, and felt as though they were in a steamer. These excessive physical burdens significantly reduced the time that HCP in PPE spent taking care of patients and increased the physical demands of their work.

Safety glasses/goggles fogged frequently, limiting visibility. Wearing an N95 respiratory protective mask with safety glasses/goggles further exacerbated fogging and restricted visibility. Poor visibility and hearing in PPE led some HCP to feel anxious or afraid, and some HCP had topical allergic reactions resulting from PPE use.

### 3.3 A comprehensive summary of factors affecting PPE use

**Figure 1:**
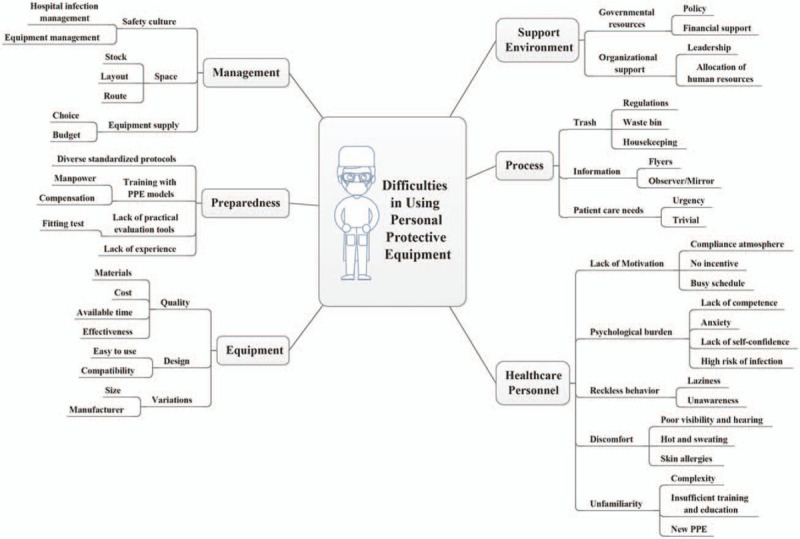
A mind map presenting a comprehensive summary.

Several other points affecting PPE use except 6 themes mentioned above were concluded in a mind map, including support environment, management, process, preparedness, HCP, and equipment (Fig. 1). Due to different sources of PPE, either allocated by the government or donated by the public, the quality of the PPE was different, and the individual compliance with recommendations for PPE use was influenced by the variability of this quality. Moreover, inadequate PPE supplies influenced its use as well. Limited hospital layouts interfered with space management for PPE use. Inappropriate positioning of devices in the environment created hidden dangers to HCP. Different interpretations of organizational processes and PPE guidelines led to confusion. A lack of training with actual PPE items was a great challenge encountered by HCP in workplaces at high risk of 2019-nCOV transmission, due to insufficient PPE supply. PPE use was also affected by a variety of potential factors, including task characteristics and lack of incentives for HCP compliance.

## 4 Discussion

Our findings provide insights on how to improve PPE use in hospitals. Improper use of PPE and PPE malfunctions causes self-contamination during patient care and in the doffing process, which may lead to HCP infection. One study showed that only 34% of subjects showed correct adherence in PPE use with variability by provider type.^[5]^ Identification of the types of errors that occur in PPE use is a vital step toward determining a solution. Researchers reviewed 325 HCP events for compliance to precautions and found 283 failures. Of these failures, 102 cases were violations of safety practices or procedures, such as entering wards with incomplete coveralls; 44 cases were process errors, such as being interrupted when removing PPE; and 37 lapses were pointed out, such as accidental self-contact without realizing it.^[6]^ Those mistakes can lead to pathogen transmission. A study showed that up to 37% of HCP's hands were contaminated after taking off contaminated gloves, but the severity of the contamination differed depending on the doffing skills.^[7]^ Another simulation study on doffing of gloves and coveralls showed that 46% of subjects had self-contamination of skin or clothing, which is extremely difficult to identify in the real world.^[8]^

Investigators noted that the design shortcomings of PPE had negative effects on PPE practices. Some scholars believe that human factor engineering is a promising way to solve some of these problems.^[9]^ Different designs of PPE were evaluated by investigators, such as convenience features for the doffing of gloves, designs that help in the removal of items from around the neck, improved fasteners for easy wear and removal, and color coding to help distinguish clean sides and potentially contaminated areas.^[10–12]^

Five key characteristics of the environment layout that can improve safety during doffing of complex PPE items were proposed by investigators for HCP: promotion of communication in doffing areas, visual cues for contaminated areas, stability devices to help with balance in doffing areas, promoted automatic safety selection in doffing areas, and context awareness provided by the environment.^[3]^ Researchers have also implemented an optimized doffing area design in a simulated environment with simple interventions, such as chairs or grab bars for balancing, delineating contaminated zones on the floor, and using mirrors to reduce risky behavior during doffing.^[13]^ Similarly, investigators from other Prevention Centers have shown that a series of interventions, including team building, PPE selection, doffing procedures, training, and the built environment, can effectively reduce the risk of self-contamination in practice.^[14]^

It is necessary to know the ability and effectiveness of PPE in protecting HCP as well as the quantification of transmission risk. Studies on transmission dynamics and PPE effectiveness so far have relied on the use of safe surrogate markers that are valuable tools for identifying and quantifying the potential risks of pathogen transmission for training and research.^[8]^ Some fluorescent markers with ultraviolet light either alone or in combination with other surrogates have been utilized to assess the ability and effectiveness of PPE. For instance, investigators evaluated the use of fluorescent markers combined with polystyrene latex spheres to quantify potential inhalation exposure.^[15]^

No matter what type of work task is going to be performed, any education and training should not only focus on “how” to use PPE, but it should also focus on “why” this equipment is necessary. Given that some HCP in the study questioned the amount of protection provided by PPE, “why” may be particularly important. For instance, some participants said that they believed gloves can protect them from a causative agent, so hand hygiene was superfluous. However, another study showed that doffing of PPE was often incorrect. HCP will often inadvertently touch the contaminated environment on exit once PPE was completely removed.^[16]^

There are several limitations associated with this study. Although stratified random sampling scheme was utilized to recruit participants to ensure that varied experiences were represented in the study, some individuals with unique experiences on issues of PPE use declined to recruitment efforts due to personal reason, which may bring an omission in our study. In addition, HCP enrolled in the study were from a hospital with the relatively small sample size that generalizations to other health care settings may be limited. Further large-scale research is needed to explore the problems of PPE use encountered by health care personnel. Therefore, the design and production of high-quality and suitable PPE products with robust evidence is essential for ensuring HCP and patient safety in future epidemic outbreaks. Moreover, intensive education and training will contribute to increasing competencies of HCP in PPE use.

## Acknowledgments

The authors thank the 20 participants for their generous contributions.

## Author contributions

XG and YJ designed this study. SL revised this manuscript and answer reviewers’ questions point by point. KH, XC, and YQ played major roles in the information collection and collation. JF and HY finished the manuscript. QX was responsible for modifying the language. All authors reviewed and approved the final version of the manuscript.

**Conceptualization:** Ying Jiang, Xin Gou.

**Data curation:** Xiao Chen, Qian Xu, Yujiao Qi.

**Formal analysis:** Hubin Yin.

**Investigation:** Kaihui Hu.

**Project administration:** Simin Liang.

**Resources:** Kaihui Hu.

**Writing – original draft:** Jing Fan.

**Writing – review & editing:** Simin Liang.
